# Intraneural Ganglion of the Peroneal Nerve—A Rare Cause of Pediatric Peroneal Nerve Palsy: A Case Report

**DOI:** 10.1055/s-0042-1742608

**Published:** 2022-03-10

**Authors:** Florian Bucher, Vincent Maerz, Doha Obed, Peter M. Vogt, Birgit Weyand

**Affiliations:** 1 Department of Plastic, Aesthetic, Hand and Reconstructive Surgery, Hannover Medical School, Hannover, Germany; 2 Medizinische Hochschule Hannover - Klinik für Plastische, Hand- und Wiederherstellungschirurgie, Hannover, Germany

**Keywords:** intraneural ganglion, peripheral neuropathy, peroneal nerve palsy

## Abstract

Intraneural ganglia are benign mucinous cysts located within the epineurium of a peripheral nerve. The pathogenesis and formation of intraneural ganglia are controversial. The main theories described in the literature are of degenerative, synovial or de novo occurrence. We present the case of a 14-year-old boy who presented in our outpatient clinic with a complaint of interdigital neuralgia between hallux and second toe, as well as left foot drop. MRI examination showed a hyperintense cystic distension of the common peroneal nerve measuring 130 mm × 5 mm extending from the poplitea to the anterior compartment of the leg. We performed microscopic decompression and neurolysis surgery. The cyst showed a sac-like distension at its distal end with connection to the tibiofibular joint and was resected. After 8 weeks, postoperatively, the boy claimed to be pain-free and slight recovery of the superficial peroneal nerve was noticed. At 6 months postoperative, the patient showed a continuous improvement of motor function, demonstrating foot eversion with 3/5 muscle strength and foot extension with 2/5 muscle strength. Intraneural ganglia reported for pediatric patients represent a very rare entity. To the best of our knowledge, less than 15 cases have been described within the English-speaking literature.

## Introduction

Intraneural ganglia are nonneoplastic fluid-filled cysts located within the epineurium of peripheral nerves. 1 They mostly affect the common peroneal nerve, while radial and ulnar nerves are rarely involved. Peroneal intraneural ganglia are most frequently found in middle-aged men. 2

The pathogenesis of intraneural ganglia is not fully understood yet. Many reports state a de novo occurrence, while Spinner et al proposed a “synovial theory.” 3 4 Based on imaging, operative, and histological findings, Spinner et al postulated that a degenerative joint was causing a pathological connection to an articular nerval branch. The cyst would then further extend along the way of least resistance. The dimensions and distribution of intraneural ganglia would then be determined by complex pressure fluxes. 3 4

Peroneal nerve ganglia can result in peripheral nerve compression leading to motor deficits and peripheral neuropathy. Most commonly patients present with a foot drop with subsequent high stepping gait and associated sensory deficits. 5 6

Magnetic resonance imaging (MRI) and nerve ultrasonography are most frequently used to visualize the cysts and to gain information about their expansion. 7

We hereby describe the case of a 14-year-old boy presenting with left drop foot caused by a large intraneural ganglion of the peroneal nerve.

## Case Presentation

A 14-year-old boy presented in our outpatient clinic with a complaint of interdigital neuralgia between the hallux and second toe, as well as left drop foot. He and his family estimated the onset of symptoms approximately 6 months ago. Due to the severe acute respiratory syndrome (SARS-CoV-2) pandemic, the boy was unable to meet with peers to perform sport activities and spent most time indoors, paying little attention to the symptoms. Previous trauma was denied. Whether the onset of symptoms was sudden or progressive could not be recalled. Aside from the symptoms of left drop foot, the boy was healthy and did not otherwise present any other comorbidities.

A neurological examination was performed approximately 5 months after the onset of symptoms and revealed profound motor weakness of the peroneal nerve. Nerve conduction velocity was performed and showed that no conduction velocities were derived from the left peroneal nerve. Ultrasound examination highlighted a cystic distension of the peroneal nerve at the level of the knee joint extending proximally in the lower thigh.

**Fig. 1:**
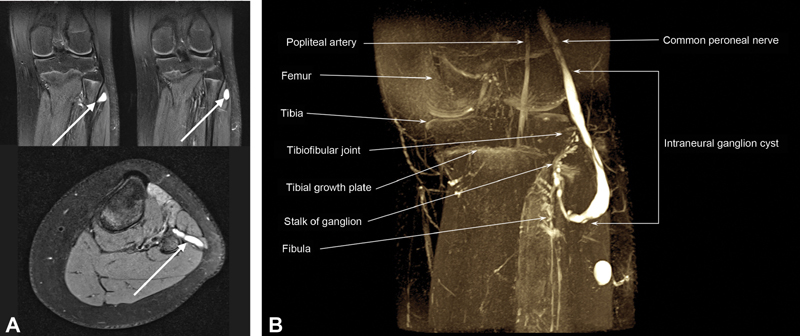
**A** ) Magnetic resonance imaging of the left knee indicating an intraneural cystic ganglion of the left common peroneal nerve in frontal and coronal plane. arrows indicate the intraneural ganglion in frontal and coronal plane. **B** ) Magnetic resonance imaging of the left knee with three-dimensional reconstruction of the intraneural ganglion of the left common peroneal nerve.

MRI (Siemens Magnetom Avanto 1.5 Tesla) was performed to assess for any structural abnormalities of the left lower limb. T2-weighted images showed a hyperintense cystic distension of the common peroneal nerve measuring 13 cm with a diameter of 5 mm extending from the poplitea to the anterior compartment of the leg. No abnormalities were described regarding the knee joint ( Fig. 1A and B ).

The boy was urgently referred to our special consultation hour for peripheral nerve surgery. On clinical examination, the boy presented a profound left foot drop. Motor testing demonstrated 0 of 5 muscle strength for extensor hallucis longus and extensor digitorum longus, resulting in impairment of foot eversion and foot dorsiflexion. Interdigital dysesthesia was described between the hallux and second toe corresponding to the dermatome innervated by the deep peroneal nerve. Physiological deep tendon reflexes were elicited for the Achilles tendon, patellar reflex, and tibialis posterior muscle tendon. The patient had a positive Tinel's sign at the level of the fibular neck radiating into the calf.

**Fig. 2:**
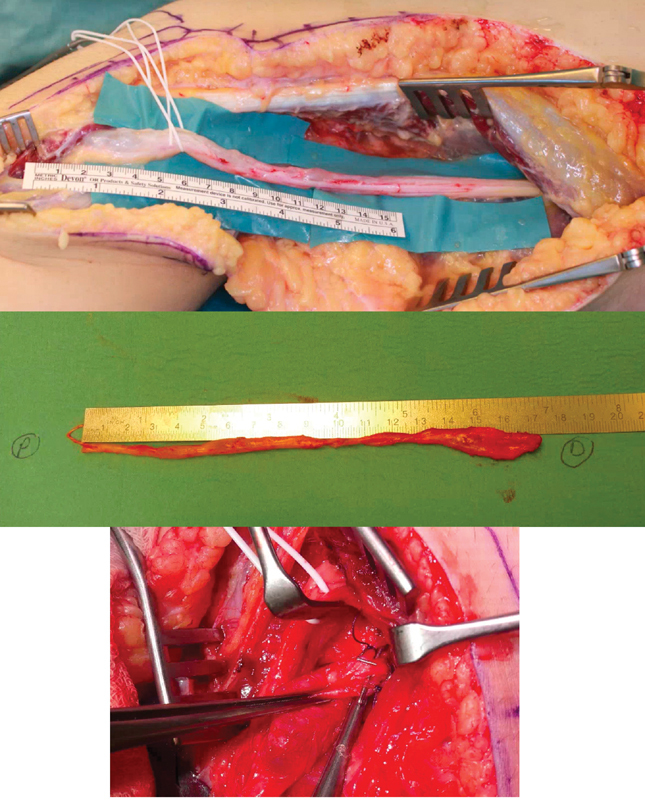
Intraoperative view of the dissected left peroneal nerve (top), after microsurgical decompression and clipping (middle), and clipped articular branches of the cyst (bottom).

We performed surgical decompression and neurolysis under general anesthesia. The common peroneal nerve was dissected and showed a distended irregular cystic, sausage-like shaped tumor over 17 cm in length from the sciatic nerve proximally to the tibial nerve distally. The epineurium was incised and careful nerve decompression was performed microscopically. The cyst showed a sac-like distension at its distal end with connection to the tibiofibular joint ( Fig. 2 ). It contained clear gelatinous fluid and was sent for histopathological examination.

On the first postoperative day, the boy described interdigital pain relief with preserved sensation while the motor function remained unchanged.

The patient was discharged with an electrical peripheral functional muscle stimulator and returned 8 weeks postoperative for follow-up examination. The boy claimed to be pain-free and reported normal sensation for the affected foot. Slight recovery of the superficial peroneal nerve was noticed as motor testing demonstrated 2/5 muscle strength for eversion of the affected foot, although 0/5 muscle strength persisted for foot extension, consequently leading to a steppage gait. The patient had a negative Tinel's sign at the level of the scar and the fibular neck and physiological deep tendon reflexes were elicited for the Achilles tendon, patellar reflex, and tibialis posterior muscle tendon. The boy received a static foot drop splint and continued using the electrical peripheral functional muscle stimulator.

The patient returned to our consultation hour 6 months postoperatively. He still presented a steppage gait. However, motor function showed a continuous improvement as eversion of the affected foot demonstrated 3/5 muscle strength. The patient was now able to extend the affected foot demonstrating 2/5 muscle strength. The Tinel sign was positive at the level of the proximal scar and along the nerve's anatomical course up to the ankle, demonstrating a tendency toward recovery for peroneal nerve tissue. Furthermore, the boy complained about dysesthesia of the dermatome innervated by the deep peroneal nerve. The slow but steady progress was noted and the patient will return for follow-up within 12 months postoperative.

## Discussion

Peroneal nerve palsy is the most common neuropathy of the lower limb. Various etiologies are described in the literature, while compression is the most common cause. 8 Peroneal nerve palsy accounts for 15% of mononeuropathies in adults. 9 Pediatric mononeuropathies are extremely rare findings. 10 Epidemiologic studies regarding the incidence of pediatric peroneal nerve palsy could not be retrieved in the literature.

Intraneural ganglia are benign mucinous cysts located inside a peripheral nerve with a connection to the adjacent joint. The peroneal nerve is the most common location for intraneural ganglia. 11

The pathogenesis and formation of intraneural ganglia is controversial. The main theories which have been described in the literature are of degenerative, synovial, and tumor origin. 12 However, the proposed theories often fail to explain cases published in the literature. Spinner et al performed intraneural dye injection and postulated a unifying articular (synovial) theory. 4 Three sequential phases were described depending on the dynamic course of the injected dye. During primary ascent, proximal extension from the superior tibiofibular joint in one or more layers of epineurium occurs followed by a cross-over within the sciatic nerve. Ultimately, terminal branch descent down the terminal branches of sciatic nerve was highlighted.

Furthermore, a complex relationship between pressure, fluxes, and dynamic appearance in relation to cysts was identified. 4 The proposed unifying articular theory by Spinner et al was supported by the Desy et al in 2016 who performed a systematic review including magnetic resonance imaging (MRI) findings of intraneural ganglion cysts. 13

Intraneural ganglia reported for pediatric patients represent a very rare entity. To the best of our knowledge, less than 15 cases have been described within the English-speaking literature.

Symptoms are nonspecific and include pain and motor/ and or sensory deficits. 14 Electromyographies and nerve conduction studies can be used to quantify peripheral nerve lesions. Imaging studies, such as computed tomography (CT), ultrasound, or MRI, provide further input about the size and localization of cysts preoperatively. 15

Surgical nerve decompression and cyst resection are recommended as the treatment of choice in the literature. Spinner et al proposed the following treatment algorithm: nerve dissection, disarticulation of the tibiofibular joint, decompression of the cyst, and articular branch disconnection. 12 Lateur et al proposed that an arthrodesis of the proximal tibiofibular joint with partial resection of the fibula had the lowest complication rate. 16 However, we find this approach unsuitable for pediatric patients. In the presented case, the nerve was carefully dissected under microscope magnification. As proposed by Spinner et al, 3 articular branches were disconnected upon cyst decompression. However, disarticulation of the joint was not performed as we considered this traumatic procedure potentially harmful to the epiphyseal growth plate and not suitable for a pediatric patient. On the first postoperative day, the boy already described interdigital pain relief with preserved sensation. The foot drop remained unchanged until discharge on the third postoperative day. Follow-up visits in our surgical consultation for peripheral nerves are generally scheduled after 6 weeks, 6 months, and 12 months postoperatively. During the latest follow-up, the patient demonstrated good improvement of motor function after surgical decompression and neurolysis, highlighting the fact that functional improvement can be achieved even for patients with delayed presentation.

When looking at the literature, overall recovery after nerve decompression appears to be promising. Consales et al described a pediatric case with a large 20-cm intraneural ganglion of the common peroneal nerve with ipsilateral foot drop. During follow-up at 26-months later, complete motor function restoration was noticed. 14

The case series by Akcakaya et al also showed an improved outcome following microsurgical decompression. However, two out of three patients with affection of the peroneal nerve had a preserved motor function preoperative which improved during the follow-up. 5

## Conclusion

We postulate late presentation to the surgeon after onset of symptoms, remaining preoperative motor and sensory functions, as well as size of intraneural ganglia as potential indicators for long-term outcome. However, large case series are needed to reach statistically significant results. When looking at the current studies published in the literature, a follow-up of >24 month seems reasonable. In terms of long-term restoration of function, we are making a favorable prognosis. As the patient already showed gradual restoration of motor function during a 6-month follow-up, a full functional restoration during long-term follow-up can be expected. Being a specialized center for peripheral nerve surgery, we noticed higher recovery potential in pediatric patients even if paresis was present preoperatively. However, a fast presentation to the surgeon seems to be the keyhole for excellent postoperative results.

## Notes

New Insights and the Importance for the Pediatric Surgeon Intraneural ganglia of the common peroneal nerve represent a rare finding causing nerve compression symptoms with palsy and sensory deficits in pediatric patients. Early diagnosis and surgical treatment play an important role in motor and sensory outcome. Being easily accessible for diagnostic imaging by ultrasound or magnetic resonance imaging, intraneural ganglia should be considered in the differential diagnosis of pediatric drop foot.
